# Antimicrobial stewardship curricula for undergraduate healthcare education applicable to the UK: a full review of online resources

**DOI:** 10.1093/jacamr/dlaf154

**Published:** 2025-09-23

**Authors:** Naomi Fleming, Sandra J Martin, Antonella P Tonna, Mamoon A Aldeyab, Ryan A Hamilton, Sally Tipping

**Affiliations:** NHS England, London, UK; School of Pharmacy and Medical Sciences, University of Bradford, Bradford, UK; School of Pharmacy, Applied Sciences and Public Health, Robert Gordon University, Aberdeen, UK; Department of Pharmacy, School of Applied Sciences, University of Huddersfield, Huddersfield, UK; Reading School of Pharmacy, University of Reading, Reading, UK; School of Pharmacy, De Montfort University, Leicester, UK; Pharmacy Department, The Royal Devon University Healthcare NHS Foundation Trust, Exeter, England

## Abstract

**Background:**

Antimicrobials are the cornerstone of modern medicine, used to treat millions of people worldwide. Antimicrobial stewardship (AMS) is included as part of healthcare professional (HCP) undergraduate (UG) curricula; however, these have not been compared with WHO guidance and it is not known if there are education resources to help embed these principles into UG curricula and whether content may vary.

**Aims:**

To identify published UG curricula or competency frameworks (CFs) for AMS relevant to medical, nursing, pharmacy, dental and allied HCPs in the UK. Also, to assess whether these curricula meet WHO recommendations and identify any gaps compared with the UK Health Security Agency (UKHSA) antimicrobial prescribing and stewardship competency framework.

**Methods:**

A search for online education resources was carried out to identify UG curricula or CFs for HCPs for AMS or antimicrobial resistance applicable to the UK.

**Results:**

Seven curricula or CFs were identified and reviewed. One AMS CF for all UG health workers, three for pharmacy UG students, one nursing AMS CF, one medical AMS CF, and the pathology curriculum. Domains varied between them but significant overlap was identified, and the majority of knowledge objectives in the WHO modules were covered in all UG curricula/CFs (except those objectives listed under the role of laboratory staff, which were only in the pathology curriculum). Gaps were identified between the most recently published UG curricula for pharmacists and the UKHSA antimicrobial prescribing and stewardship CF.

**Conclusions:**

There is significant correlation between the UG curricula/CFs for HCPs in the UK, the WHO curricula guide, and UKHSA antimicrobial prescribing and stewardship CF; however, there are some identified gaps. Despite the availability of UG curricula and/or CFs for the majority of HCPs, these are not consistently embedded in UG training.

## Introduction

Antimicrobials are the cornerstone of modern medicine, used to treat millions of people worldwide.^1^ They are used to treat infections in humans and animals and in support of surgery and modern cancer therapies. Organisms that become resistant to antimicrobials make treatments less effective, causing harm to humans and animals. Resistant organisms spread through people, animals, food and the environment, creating a major public health threat.^1^ Antimicrobial stewardship (AMS) is defined as ‘an organizational or healthcare system-wide approach to promoting and monitoring judicious use of antimicrobials to preserve their future effectiveness.’^2^ It consists of a combination of interventions that monitor antimicrobial use and resistance in microbes, the development of new drugs, treatments and diagnostics, individuals’ behaviour relating to infection prevention and control, and healthcare professionals’ prescribing decisions.^2^

Core drivers for this scoping review on AMS undergraduate (UG) curricula for healthcare professionals (HCPs) are the UK 5 year national action plan (NAP) for antimicrobial resistance (AMR) 2024–2029,^1^ the updated 2023 UK Health Security Agency (UKHSA) antimicrobial prescribing and stewardship competency framework (CF),^3^ and the 2019 WHO health workers’ education and training on antimicrobial resistance curricula guide.^4^ The UK NAP contains the commitment: ‘We will further embed, and will require, the completion of appropriate infection prevention and control (IPC) and AMS training for all health and social care workers and students, to support implementation of best practice for IPC and AMS in their setting and, for specialist posts, to provide career pathways to promote skills retention and succession planning.’^1^ It goes on to state: ‘Embedding best practice within the curriculum, at undergraduate and post-graduate level, of those in frontline health and social care services is critically important. Ensuring that training programmes instil not just theoretical knowledge but hands-on experience in implementing IPC and AMS measures will improve IPC, infection management and antimicrobial stewardship and build on the already substantial common awareness of these issues in the health and care community.’^1^ The NHS England Antimicrobial Resistance (AMR) and Antimicrobial Stewardship (AMS) Indicative Curriculum for pharmacy UGs is the most recently published.^5^

### Aims

The aims of this review were to understand whether published UG CFs for HCPs in the UK are easily accessible, whether they meet WHO recommendations and contain the competencies needed to support prescribing, or whether there are gaps in curricula that need to be addressed as part of the NAP.

## Methods

This scoping review followed the five-stage framework outlined by Arksey and O’Malley to answer a research question.^6^

### Stage 1: Identifying the research question

This review aimed to identify UG curricula and or CFs for AMR and AMS for HCPs. The main research question was, ‘What is the published literature on UG curricula or CFs for AMR or AMS for medical, nursing, pharmacy, dental or generalist HCPs applicable to the UK?’

Supplementary questions were: ‘Are they easily accessible? Do they meet WHO recommendations and are there gaps in the most recently published CFs for UG curricula compared with the updated UKHSA antimicrobial prescribing and stewardship CF?’

### Stage 2: Identifying relevant studies

The literature search comprised two phases:

1. Initial search for policy documents from the UK and WHO on education, including WHO, NHS England (NHSE), Health Education England (HEE), NICE, UKHSA and the Royal College of Nursing (RCN).
2. Online searches of databases for academic papers using: MEDLINE, Embase and CINAHL. Keywords included ‘undergraduate’, ‘competency framework’, ‘students’, ‘nursing’, ‘medical’, ‘dental’, ‘antimicrobial stewardship’, ‘antimicrobial resistance’. Bibliographies of resources identified by the literature search were searched for potential new resources.

### Stage 3: Study selection

**Figure 1.**
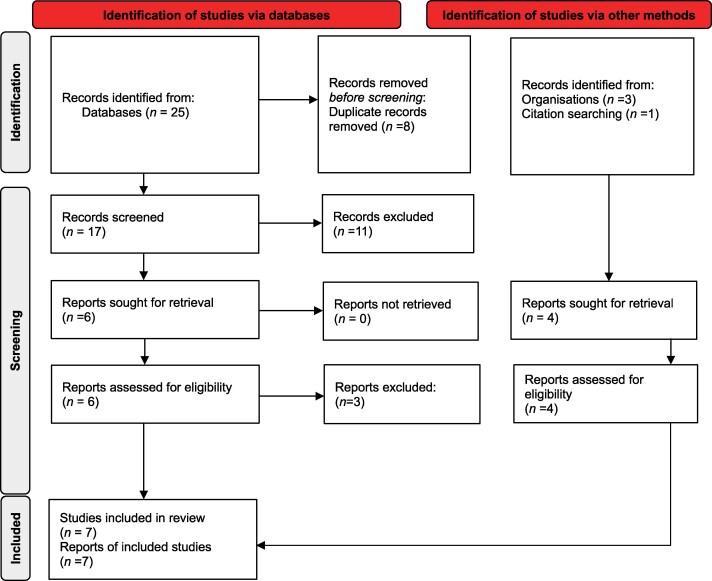
Online education resource identification and selection processes. Source: Page MJ *et al. BMJ* 2021; **372**: n71. https://doi.org/10.1136/bmj.n71. This work is licensed under CC BY 4.0. To view a copy of this licence, visit https://creativecommons.org/licenses/by/4.0/.

Researcher N.F. screened titles, abstracts and content from the literature searches based upon inclusion and exclusion criteria. Resources were included if they were available as open access or available through authors’ institutions, written in English and published between January 2014 and November 2024. No resources were excluded due to access issues. Resources were excluded if they were not UG inclusive and did not answer the research question. See Figure 1 for details of studies and the selection process based on methodology by Page *et al.*^7^

### Stage 4: Charting the data

**Figure 2.**
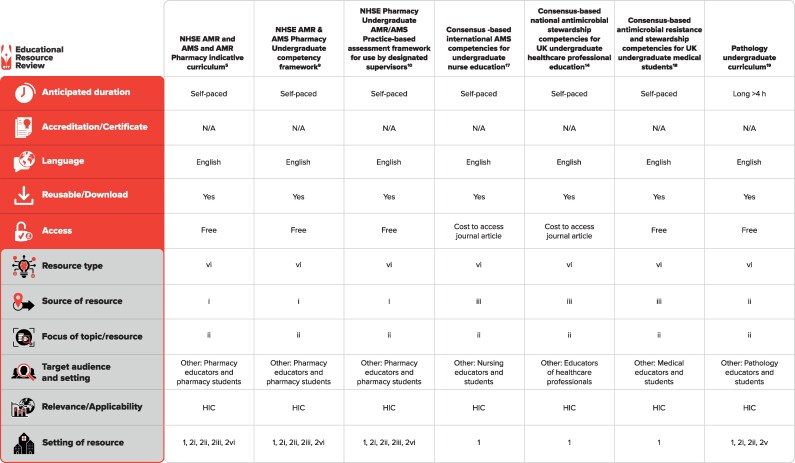
Characteristics of UG curricula/CFs included. Resource type: (i) website online reading material and other resource; (ii) website primarily aimed at news items; (iii) online/distance learning courses [massive open online courses (MOOCs), unfacilitated courses, online modules]/community of practice; (iv) webinars, video, online lectures (including PowerPoint), podcasts, animation video, maps, photos; (v) clinical practice AMS materials: pdfs, PowerPoints, newsletters, infographics, pamphlets, e-portfolios, workbooks; (vi) guidelines, policies, handbooks; (vii) material from face-to-face non–e-learning courses (programmes, teaching materials, etc. from workshops, lectures, seminars); (viii) e-books via apps; (ix) public, media, political awareness and engagement materials; (x) commercial advertising (TV, radio, film, social media); (xi) evidence: by systematic reviews/meta-analysis in relation to AMR; (xii) datasets, compelling/illustrative case studies on AMS; (xiii) patient stories. Source of resource: (i) governments; (ii) professional societies; (iii) universities/higher education institutes; (iv) healthcare facilities (hospitals, GPs, clinics, healthcare centres); (v) WHO; (vi) industry; (vii) insurance companies; (viii) non-governmental organizations. Focus of topic/resource: (i) principles/practice of prudent prescribing; (ii) AMS principles/practice; (iii) guidelines/policies/pathways for syndrome management of infections (empiric organ or microorganism specific); (iv) infection prevention/control; (v) implementation/behaviour change; (vi) evaluation/measurement; (vii) evidence gathering (systematic review, etc.). Setting of resource: 1. Pre-service (university, higher education institution); 2. Service: (i) hospital; (ii) outpatient clinic; (iii) community/general practice; (iv) long-term care facility/nursing home; (v) hospital and ambulatory; (vi) others. The full classification scheme is available at http://bsac.org.uk/wp-content/uploads/2019/03/Educational-resource-review-classification-scheme.pdf. HIC, high-income countries; NA, not applicable.

Full texts of identified resources for inclusion in the review were synthesized and analysed, and data extracted using a customized data charting form (see infographic Figure 2).

### Stage 5: Collating, summarizing and reporting the results

A narrative account of the charted data was produced, which included the distribution of studies geographically, range of interventions, research methods and outcomes.

## Results

The search strategy identified a total of 29 resources, of which 7 met the inclusion criteria for final review. Resource identification and selection processes are detailed in Figure 1.

Education resources identified were summarized in an infographic (Figure 2). Some resources were easily accessible whereas others were papers that required payment, limiting usability; however, all were included in this review.

### WHO health workers’ education and training on antimicrobial resistance: curricula guide

**Figure 3.**
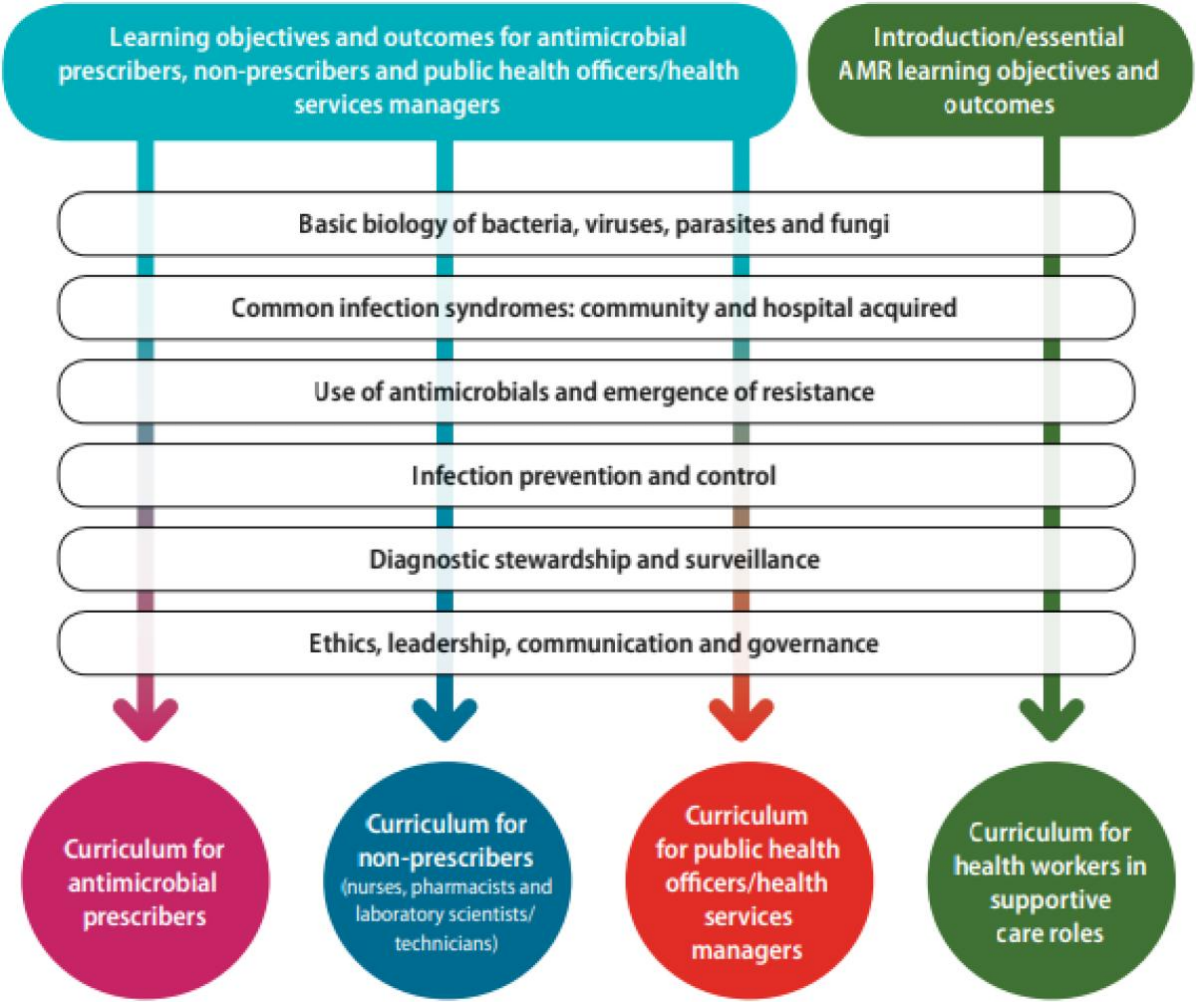
WHO matrix model for antimicrobial resistance curricula.^4^

In 2017, the WHO launched the ‘Competency Framework for Health Workers’ Education and Training on Antimicrobial Resistance’,^8^ developed through a combination of a review of global AMR competencies, mapping of education and training resources, and consultation with experts from the WHO and AMR research and professional organizations. The goals of the CF are to promote standardization in quality and ensure coverage of educational initiatives and resources.^8^ Building on the foundation of the WHO CF, a curricula guide for educators of HCPs outlines learning objectives and expected outcomes. To enhance its relevance and applicability within interprofessional settings, the guide’s flexible modular structure simplifies the selection and translation of content into teaching syllabuses and learning materials based on local priorities and needs.^4^ Five modules are aligned with the WHO CF and structured across four categories of health workers in a matrix model (see Figure 3). Modules are: foundations that build awareness of AMR; appropriate use of antimicrobial agents; IPC; diagnostic stewardship and surveillance and ethics; leadership; and communication and governance.

Each module contains a competency statement and learning objectives that are divided into required knowledge, skills and attitudes. This curriculum guide contains recommendations for teaching methods, recommends incorporating multiple pedagogical and assessment techniques, and guidance on how to review current AMR curricula. It is consequently ideal as a reference tool against which other curricula may be mapped.

Production through the consensus of international experts increases credibility and widens applicability to support education and training for healthcare personnel in all countries; inclusion of assessment methods and pedological methods are a strength. Limitations include that the objectives are aimed at UG and postgraduate health education and training institutions without differentiation between them.

Modules from the WHO have been compared with UK-applicable CFs and curricula (matching similar domains where possible). Numbers of knowledge objectives across prescribers in the WHO modules or number of competencies for each domain within the UK-applicable CFs have been added except for the Royal College of Pathologists, where domain numbers have been added (Table 1).

**Table 1.** Comparison of structure of the profession-specific frameworks against WHO and UKHSA structures

	WHO^4^ 5 modules, 75 knowledge objectives for prescribers	UKHSA^3^ AMS prescribing competencies 6 domains, 113 competencies	UK^9^ pharmacy UG 6 domains, 74 competencies	UK nurses UG^10^ 6 domains, 63 competencies	International HCP^11^ UG 6 domains, 55 competencies	UK medical^12^ UG 6 domains, 58 competencies	Royal College of Pathologists^13^ UG curricula 21 domains, 308 competencies
Domain 1	Foundations that build awareness of antimicrobial resistance (26)	Antimicrobial resistance and antimicrobials (16)	Antimicrobials and antimicrobial resistance (8)	Antimicrobials and antimicrobial resistance (5)	Antimicrobials and antimicrobial resistance (5)	Antimicrobials and antimicrobial resistance (5)	Infection and its treatment Microbiological, biochemical, histopathological, immunological knowledge and skills (5)
Domain 2	Appropriate use of antimicrobial agents (28)	Antimicrobial stewardship principles (16)	Antimicrobial prescribing and stewardship (24)	The diagnosis of infection and the use of antimicrobials (17)	The diagnosis of infection and the use of antimicrobials (14)	Antimicrobial prescribing and stewardship (20)	Knowledge and understanding of pathology and the role it plays in diagnosis and therapy Infection and its treatment: Molecular pathology Digital pathology Pathophysiological knowledge sufficient to reach a diagnosis and begin treatment (6)
Domain 3	Infection prevention and control (7)	Infection prevention and control (29)	Infection prevention and control (19)	Infection prevention and control (18)	Infection prevention and control (16)	Infection prevention and control (18)	Professional values and behaviours across subspecialties (1)
Domain 4	Diagnostic stewardship and surveillance (7)	Monitoring, learning and interprofessional collaborative practice (12)	Interprofessional collaborative practice (10)	Interprofessional collaborative practice (6)	Interprofessional collaborative practice (6)	Interprofessional collaborative practice (5)	Professional values and behaviours across subspecialities Diagnosis and medical management Using information effectively and safely Healthcare service organization as relevant to pathology (4)
Domain 5	Ethics, leadership, communication and governance (7)	Person-centred care (8)	Person-centred care (8)	Person-centred care (7)	Person-centred care (5)	Person-centred care (5)	Communication and interpersonal skills General organization of immune system Immunodeficiency (3)
Domain 6		Prescribing antimicrobials (32)	Vaccine uptake (5)	Antimicrobial prescribing practice (10)	Antimicrobial prescribing practice (9)	Vaccine uptake (5)	Prescribing medication safely Basic knowledge of current vaccines for prevention of infection (2)

(n) is the number of competencies in this domain.

### NHSE initial education and training of pharmacists

NHSE initial education and training of pharmacists comprises the following elements: (i) AMR and AMS indicative curriculum,^5^ (ii) AMR and AMS pharmacy UG CF,^9^ and (iii) pharmacy UG AMR/AMS practice-based assessment framework for use by designated supervisors.^14^

NHSE has published these three resources to be used together. Their use is not compulsory, but they are a guide to support educators at higher education institutes in England to ensure that student pharmacists gain the appropriate skills required to practise AMS effectively. The indicative curriculum was developed by NHSE workforce education and training and antimicrobial prescribing and medicines optimization teams, the National Antimicrobial Pharmacy Education Group and the Pharmacy Schools Council. It contains six domains: IPC; antimicrobials and AMR; antimicrobial prescribing and stewardship; vaccine uptake; person-centred care; and interprofessional collaborative practice. Each domain has a competency statement alongside key themes and appropriate learning resources for that domain. Domains are mapped to the General Pharmaceutical Council (GPhC) learning outcomes^15^ and Royal Pharmaceutical Society (RPS) prescribing competencies^16^ to indicate how it aligns with initial education and training of pharmacist (IETP) standards. Strengths are that it has been developed through the consensus of AMS education experts, is specifically tailored to undergraduate pharmacy education, maps to IETP learning outcomes, and provides suggested learning resources for educators and students. One of these signposted resources is the British Society for Antimicrobial Chemotherapy (BSAC) Keep Antibiotics Working (BSAC-KAW),^17^ which hosts educational resources on AMR and AMS aligned with the six domains. It can be accessed by educators, healthcare professionals and students, and contains resources in different pedagogical formats, including articles, websites and animations supporting different learning styles.

Limitations include variable implementation across individual pharmacy schools as it is not compulsory. The resources are published by NHSE; however, they were developed in conjunction with English, Welsh, Scottish and Northern Ireland schools of pharmacy. Although developed for students who will be pharmacist prescribers, it has yet to be evaluated with qualifying pharmacists.

The NHSE ‘AMR and AMS pharmacy undergraduate competency framework’ was adapted from existing published AMS CFs in the UK^11,18,19^ and tailored to meet the requirements of UG pharmacy students for the new IETP. It contains the same six domains and domain-level competency statement as the curriculum; however, it is more detailed and provides a comprehensive CF with accompanying descriptors for each domain.

Strengths include that it has been developed through consensus of AMS education experts, it is specifically tailored to UG pharmacy education, and is a detailed framework of all competencies required in the different domains. Limitations are that it is an extensive framework with 74 descriptors, which will be challenging to deliver in the already busy IETP, and it has variable implementation as it is not compulsory.

To support the NHSE pharmacy UG AMS curriculum and CF, a separate practice-based assessment framework for designated supervisors was developed.^6^ This can be used by supervisors for pharmacy students and pre-registration foundation pharmacists on clinical placements in any healthcare setting. This contains theoretical and practical assessment suggestions for the six domains.

The benefits of this resource are that it was developed through consensus by experts in AMS pharmacy education; educators and supervisors can use it to plan AMS-related educational activities and methods to assess student learning. Moreover, students can retain evidence from educational activities and assessments as part of practice-based learning, for example, in a portfolio. Disadvantages are that educators and learners have to find time to undertake AMS-related activities as part of a busy, wider experiential learning, awareness of the resource and consistent implementation as it is not compulsory.

The strengths of these three resources are co-production with policy makers, educators and professional pharmacy bodies. They support implementation and benchmarking as they include mapping to pharmacy curricula, links to practical resources, detailed competency descriptors and ideas for practical assessment.

### Consensus-based international antimicrobial stewardship competencies for undergraduate nurse education

Courtenay *et al.*^10^ published a consensus-based CF in 2019. This provides international consensus on AMS competency descriptors appropriate for UG nurse education. The pre-existing UK AMS competencies appropriate for UG HCP education formed the content of the round 1 Delphi survey; the six domains are the same and include: IPC; antimicrobials and antimicrobial resistance; the diagnosis of infection and the use of antibiotics; antimicrobial prescribing practice; person-centred care; and inter-professional collaborative practice. The target audience are international UG nurse educators, regulators and professional bodies to inform proficiency standards and guidance. The strengths are: it is based on responses from an international panel of defined experts, it had a good response rate, and it offers a framework of AMS competencies appropriate for UG nurse education. Limitations include only a small number of participants in the Delphi process (15 experts) and a small number from each country, so it may not reflect the full spectrum of nurses involved in AMS. Other limitations include lack of open access to the publication and consequent implementation as this is not compulsory. It is a text document with no resources linked nor is it mapped to any existing nursing curricula.

### Development of consensus based national antimicrobial stewardship competencies for UK undergraduate healthcare professional education

This resource was the first UK CF developed by Courtenay *et al.*^11^ and published in 2018. This provides a UK consensus on a common set of AMS competencies appropriate for UG HCP education. A modified Delphi approach was used; the first Delphi round included a comprehensive list of competencies, informed by available prescribing and stewardship CFs, AMS principles, evidence of the key AMS principles delivered on UG health professional education programmes, and interprofessional competencies. The six domains are: IPC; antimicrobials and antimicrobial resistance; the diagnosis of infection and the use of antimicrobials; antimicrobial prescribing practice; person-centred care; and interprofessional collaborative practice. Fifty-five descriptors are described under these domains. The target audience for this resource are educators for HCPs in the UK (including doctors, dentists, nursing and physician associates, pharmacists, nurses, midwives and allied healthcare professionals) and regulators and professional bodies to inform proficiency standards and guidance. The strengths of this resource are that it was an interprofessional consensus process, involving a national panel of defined experts, had a good response rate, and offered a framework of AMS competencies appropriate for UG HCP education.

Limitations include that the expert panel composition does not reflect the full spectrum of professions involved in AMS, for example, dentists and doctors. Therefore, the competencies represent the views of nurses, pharmacists, physiotherapists and podiatrists. There were only small numbers of participants from each professional group, which impinges upon the generalization of results. Other limitations include lack of open access to the publication and limited implementation as this is not compulsory. It is a text document and has no resources linked, nor is it mapped to any existing healthcare curricula.

### Consensus-based antimicrobial resistance and stewardship competencies for UK undergraduate medical student’

This resource was published by McMaster *et al.*^12^ in 2020. It provides a UK consensus on competencies for AMR and AMS for UG medical education. The target audience include professional bodies, regulators, medical educators and students. It contains the same six domains as the pharmacy UG resources: IPC; antimicrobials and AMR; antimicrobial prescribing and stewardship; vaccine uptake; person-centred care; and interprofessional collaborative practice. The strengths are it is based on responses from a panel of defined experts in medical undergraduate teaching from England, Wales and Northern Ireland, it had a good response rate, and it offers a framework of AMS competencies appropriate for UG medical education. Limitations include the lack of involvement from Scotland, lack of awareness of the framework and consistent implementation as this is not compulsory. It is a text document and has no resources linked, nor is it mapped to any existing medical curricula, and it contains 58 competencies to include in a crowded medical curriculum.

### Royal College of Pathologists pathology undergraduate curriculum

This resource was published by the Royal College of Pathologists in 2019.^13^ It contains three sections on outcomes and capabilities: professional values and behaviours, professional skills and professional knowledge. Outcomes and capabilities that deal with professional knowledge are covered under broad headings with specialty-specific outcomes where applicable: the organization of pathology services; scientific principles and practice of pathology including recent advances; biomedical scientific principles as applied to general pathology; and systems of the body. Outcomes and capabilities are further divided into 21 domains with competencies listed under each domain, and delivery and assessment methods recommended for each domain. The aim of this curriculum is to align the current pathology service delivery landscape and body of evidence-based practice with the current General Medical Council (GMC) guidance to define key learning outcomes. It is intended to assist in the delivery of the curriculum by all medical schools, regardless of local variation in undergraduate curricula and their delivery. As the professional body responsible for designing training curricula, the Royal College of Pathologists has a role in setting the direction for pathology education. Strengths are that it is mapped to GMC outcomes and has a comprehensive list of competencies with suggested types of assessment and a link to e-learning resources that provide general information on pathology suitable to support undergraduate and foundation learning. There is also a link to a hub of resources for those working in microbiology, including links to upcoming continuing professional development–accredited events.

Limitations are the length and number of competencies in the curriculum. Domains that include microbiology, infection prevention and management, and AMS also include other pathology competencies, so it is not easy to separate them out.

### UKHSA national prescribing and stewardship competencies

UKHSA national prescribing and stewardship competencies were updated in 2023.^3^ The most recently published professional framework (the pharmacy UG curriculum and CF)^5,9^ was mapped to the UKHSA competencies to identify any gaps. There are 74 competencies listed in the pharmacy UGCF compared with 113 in the UKHSA prescribing competency framework, suggesting 39 competency gaps.

After scrutinizing the competencies in each of the different domains in both CFs, the majority of the 39 competency gaps were included in longer descriptors in the pharmacy UG CF; however, 16 extra competencies were identified in the UKHSA framework that were not in the pharmacy UG CF (see Table 2).

**Table 2.** Additional competencies contained within the UKHSA framework that are not present in the UG pharmacy CF

UKHSA domain	Extra competencies
Infection prevention and control	2.2. Understands and promotes the principles and practice of the prevention and control of infection^b^
	2.5. Understands that prescribing antimicrobials to patients colonized with MDR pathogens (e.g. MDR Gram-negative pathogens in urine or gastrointestinal tract) will not eradicate the pathogens and should therefore not be used as a preventive measure to stop transmission of the resistant pathogens to others
	2.16. Implements a plan that is focused on limiting cross-infection and contamination to reduce healthcare-associated infections and AMR in hospitals and community settings^b^
	2.19. Applies methods and strategies to prevent and control healthcare-associated infections, including surgical site infections, catheter-associated bloodstream and urinary tract infections, healthcare-acquired pneumonia, gastroenteritis^b^
	2.22. Promotes the decontamination and sterilization of equipment and patient areas in line with local policies and guidelines^b^
	2.25. Understands how current vaccines can benefit prescribing practices, including reducing the need for prescribing antimicrobials and decreasing antimicrobial resistant strains, e.g. *Streptococcus pneumoniae*^a^
Antimicrobial resistance and antimicrobials	3.10. Understands the basic principles of susceptibility reports (antibiograms) and other reporting tools and their interpretation
	3.12. Understands the principles of surveillance of AMR and antimicrobial use and the use of surveillance data
	3.13. Understands the mechanisms of antimicrobial resistance, including: b. the importance of selection advantages, for example the greater ability for some to colonize, to alter virulence, and how this can be an amplification process for antimicrobial resistance
	3.14. Interprets and uses AMR surveillance data where appropriate^b^
Prescribing antimicrobials	4.22. Knows about trade and generic names, and the class, of a prescribed antimicrobial to avoid possible harm to patients in whom that antimicrobial is contra-indicated, for example due to hypersensitivity, coagulopathy, or organ impairment^a^
Antimicrobial stewardship principles	5.13. Recognizes the immediate and long-term patient and ecological consequences of inappropriate antimicrobial prescription
	5.15. Prioritizes the understanding of prescribing guidelines and practical application against market incentives to proliferate the prescription of antimicrobials^b^
	5.16. Promotes capacity to search for reliable sources of unbiased/unconflicted information on the best use of antimicrobials^a^
Monitoring, learning and interprofessional collaborative practice	6.4. Uses the results of adverse event monitoring, laboratory susceptibility reports, antimicrobial prescribing audits and antimicrobial usage data to inform, in a timely manner, best antimicrobial prescribing practices, and so produces sustained improvements in the quality of patient care^b^
	6.10. Understands basic principles of behaviour change in the context of prescribing antimicrobials, how prescribing antimicrobials can be influenced by factors other than clinical need and measures to prevent this and demonstrates good prescribing habits^b^

^a^Contained within generalist pharmacy UG curricula.

^b^Practice based.

## Discussion

### Key findings

We identified seven published resources for AMS UG curricula applicable to UK HCPs. There is variation between the format and content of UG curricula and CFs, with the WHO and pathology curricula being similar in format and content, and the consensus healthcare professional CF (nursing, medical and pharmacy) being similar having developed iteratively over time from the initial 2018 Courtenay *et al.* framework.^11^

AMS education is advocated by the WHO CF for health workers’ education and training on AMR and an associated curricular guide has been developed.^4,8^ UG education and its importance as a tool to combat AMR is highlighted in the UK NAP.^1^ Herein we describe the HCP CFs and associated resources applicable to UK students. Although domains vary between the WHO modules, consensus-based healthcare professional CFs and the Royal College of Pathology curricula, there is significant overlap between them and the majority of knowledge objectives in the WHO modules are covered in all resources, except for those listed under the role of laboratory scientists/technicians, which are covered in the pathology curriculum but not the HCP curricula/CFs. Despite the World Dental Federation (FDI) policy statement ‘FDI encourages the development of educational programmes on antibiotic resistance, prescribing and stewardship that are suitable for the continuum of the professional lives of dentists and dental teams’,^20^ no UK dental-specific UG CFs were identified in the literature.

Comparing the most recently published HCP CF (for pharmacists who will be graduating as prescribers in 2026) with the updated UKHSA prescribing competencies, 16 competencies were absent (see Table 2). However, it is important to note that some of these are generic competencies that would be covered in a pharmacy UG curricula^a^ (for example: 4.22. Knows about trade and generic names, and the class, of a prescribed antimicrobial to avoid possible harm to patients in whom that antimicrobial is contra-indicated, for example due to hypersensitivity, coagulopathy, or organ impairment) and some are not possible to be taught/met until a pharmacist is in practice and will be influenced by their area of practice^b^ (for example: 6.4. Uses the results of adverse event monitoring, laboratory susceptibility reports, antimicrobial prescribing audits and antimicrobial usage data to inform, in a timely manner, best antimicrobial prescribing practices, and so produces sustained improvements in the quality of patient care).

With the updated UKHSA prescribing competencies published and with more professions being able to prescribe in the UK (including medics, dentists, nurses, pharmacists, podiatrists, physiotherapists, paramedics and optometrists) is it time for healthcare educators, policymakers and royal colleges to come together to develop one UK multi-professional prescriber-ready UG AMS/AMR curriculum with resources to aid multi-disciplinary implementation? Regulators could also play a role in ensuring AMS content and consistency. Until then, a central repository of current UG curricula, CFs and implementation resources would be useful for educators and healthcare professionals.

The common limitations of the consensus resources reviewed are their length, text-only format, lack of mapping to the profession-specific generalist education outcome frameworks, and lack of links to educational resources for educators or students to promote implementation and assessment. The initial 2018 HCP CF by Courtenay *et al.*^11^ was used iteratively for all subsequent profession-specific CF development. This has strengths in terms of consistency, but also limitations in that although methodologically sound, a relatively small number of individuals developed the initial CF. There may be selection bias, confirmation bias, attrition bias or language bias inherent in this methodology.

Two curricular resources (UG pharmacists from NHSE and pathologists from the Royal College) are mapped to GPhC and GMC learning outcomes, respectively, and contain links to educational resources for educators and students; however, it is not clear how well these are embedded into individual UG schools.

A survey by Health Education England in 2016^21^ looked at how well the previous Public Health England prescribing and stewardship competencies had been embedded into UG curricula. Forty-five universities responded, with 100 courses for the following professions: medicine, adult nursing, dentistry, pharmacy, midwifery, independent prescribing courses and allied health professionals. Respondents from 86 courses confirmed they were aware of these competencies. The average implementation rate for all universities and courses was 67% for all the dimensions, with allied health professionals, nursing and midwifery having the lowest implementation rates (41%, 46% and 52%, respectively). The domain with the lowest implementation was ‘Monitoring and Learning’. However, worryingly, ‘Prescribing antimicrobials’ and ‘AMS’ were not being taught on the majority of nursing and allied professionals courses surveyed.^21^ This is important as nurse and allied healthcare professional prescribers are increasing. The subsequent publication of the UG curricula and CFs identified in our article may have addressed this issue.

A survey of schools of pharmacy in the UK in 2022^22^ (prior to the launch of specific pharmacy UG curricula) explored the extent to which the national AMS competencies for UG healthcare education were embedded into UG pharmacy curricula; 10 schools of pharmacy responded, no institution covered all 54 AMS competencies, and 5 AMS competencies were taught at half or fewer of the institutions. It also highlighted that the course contained a lot of didactic teaching, and not a lot of delivery and assessment in practice settings.^22^ It remains to be seen whether the 74 competencies identified in the NHSE pharmacy UG CF will be embedded in the IETP programme with the resources designed to support implementation in practice-based settings. A study of the delivery of AMS competencies in UK pre-registration nurse education programmes^23^ showed that the provision of AMS teaching was inconsistent across the 35 UK universities that took part. Competencies in IPC, patient-centred care and interprofessional collaborative practice took precedence over competencies pertaining to the use, management and monitoring of antimicrobials.^23^ Barriers to implementation include accessibility of the nursing CF, and the time and capacity within courses; however, the growing risk and impact from AMR, benefits of AMS, and the fact that infection crosses many clinical pathways means that this is relevant to all prescribers and healthcare workers.

### Conclusions

We have identified seven education resources for AMS/AMR UG curricula applicable to UK HCPs with significant overlap. There is variation between the formats and contents. The WHO education curricula guide and pathology curriculum are most similar in format and content whereas the consensus healthcare professional, nursing, medical and pharmacy UG frameworks are similar, having been developed iteratively from the initial 2018 Courtenay *et al.* framework. With the updated UKHSA prescribing competencies published in 2023, the focus on UG curricula in the UK AMR NAP, and with many HCPs able to prescribe independently, healthcare educators, policy makers, regulators and royal colleges should come together to develop a mandatory UK-wide UG AMS/AMR multi-professional prescriber-ready curriculum with resources to aid implementation.
